# Regression-Based Prediction of Surface Microgeometry in Biopolymers Processed for Dental Applications

**DOI:** 10.3390/biomimetics11060375

**Published:** 2026-05-29

**Authors:** Ján Duplák, Samuel Mikuláško

**Affiliations:** Faculty of Manufacturing Technologies, Technical University of Kosice, Bayerova 1, 080 01 Presov, Slovakia

**Keywords:** biomaterials, dental implants, milling, dental crown

## Abstract

This study focuses on streamlining the manufacturing process for milling dental prosthetic components from biopolymer materials in order to achieve the best possible surface roughness. Various combinations of cutting parameters were systematically tested in experiments, and their impact on the final surface roughness of the material was analyzed. The study provides a comprehensive view of how variations in cutting speed, feed per tooth and cutting depth affect the final surface quality. The results show that the appropriate configuration of cutting parameters can significantly improve surface roughness, reducing the need for additional finishing and increasing production efficiency. The findings provide valuable information for the manufacture of polymer-based dental prosthetic components, support process optimization, and contribute to the development of accurate and reproducible computer-aided design and computer-aided manufacturing (CAD/CAM) manufacturing procedures. A full factorial design of experiments (DoE) approach was applied to evaluate the influence of cutting speed, feed per tooth, and cutting depth on the resulting surface roughness. The results confirmed that feed per tooth represented the most influential machining parameter affecting the resulting Ra values. The optimized cutting conditions resulted in the lowest surface roughness and improved process stability compared to manufacturer-recommended machining conditions.

## 1. Introduction

In recent years, biopolymers have established themselves as a promising material for the manufacture of dental prosthetic components thanks to their combination of mechanical resistance and biocompatibility. Their proper processing is key to achieving a suitable surface microgeometry, which affects not only the aesthetics and comfort of the patient but also the stability and long-term functionality of prosthetic restorations. Surface quality has a significant impact on interaction with opposing teeth, wear resistance and the need for additional modifications such as polishing or glazing. Precise specification of cutting parameters is crucial for achieving a uniform and clinically acceptable surface. Parameters such as cutting speed, feed per tooth and cutting depth can significantly affect the microstructure of the surface and the reproducibility of the process, with minor deviations leading to significant differences in the final quality of the prosthetic component. This study focuses on a systematic analysis of the influence of different combinations of cutting parameters on the surface quality of biopolymer prosthetic restorations. The main objective is to identify machining conditions that will achieve optimal surface roughness, minimize the need for additional modifications and ensure process reproducibility. The results of the experiments provide valuable insights for setting up manufacturing processes in dental prosthetic components, support the development of accurate and efficient techniques, and contribute to improving the quality and clinical effectiveness of biopolymer prosthetic components.

## 2. Theoretical Background

Digital technologies, especially CAD/CAM systems, have fundamentally transformed the manufacture of dental prosthetic components by enabling precise and efficient production with high reproducibility of shape and dimensions. Dental composite materials are increasingly used in digital prosthetic dentistry due to their favorable balance between mechanical performance, aesthetics, and machinability. Recent studies have investigated the influence of reinforcing phases on the mechanical and tribological properties of dental composites, highlighting the importance of material composition for clinical durability and surface behavior after machining. For example, the study “Fabrication, evaluation, and performance ranking of tri-calcium phosphate and silica reinforced dental resin composite materials” demonstrated that filler reinforcement significantly affects the mechanical stability and surface performance of dental composite systems. Polymer discs, including polymethyl methacrylate (PMMA), polyether ether ketone (PEEK), polyetherketoneketone (PEKK) and hybrid materials, have become key materials for temporary and permanent prosthetic restorations, as they combine low weight, bioinertness and high processing accuracy with sufficient mechanical resistance. PMMA is based on polymethyl methacrylate, while PEEK and PEKK belong to the polyaryletherketone family of high-performance thermoplastic polymers characterized by high chemical resistance, dimensional stability, and favorable mechanical properties for CAD/CAM prosthetic applications. Surface roughness R_a_ is considered an important factor influencing bacterial biofilm adhesion in the oral environment. Surface roughness parameters such as R_a_, R_q_, R_z_, and R_sm_ are commonly used for the evaluation of machined dental materials. R_a_ represents the arithmetic mean surface roughness and is the most frequently used parameter for assessing general surface quality. R_q_ describes the root mean square roughness, while R_z_ represents the average maximum height of the surface profile. R_sm_ characterizes the average spacing of surface irregularities and provides additional information about the surface topography after milling. However, the reported threshold values may vary depending on the material type, surface treatment, and experimental methodology. Conversely, several studies have reported that higher surface roughness values may contribute to increased plaque accumulation and less favorable hygienic conditions around dental restorations [1]. It is important to emphasize that this value does not come from technological recommendations but from microbiological experiments that examined the interaction between surface morphology and bacterial colonization. Although this threshold was established in earlier studies [1], subsequent modern studies [2,3] confirmed its validity for current CAD/CAM materials without re-evaluating it. More recent studies [2,3] have continued to reference surface roughness values of around R_a_ ≤ 0.2 µm in relation to the reduced bacterial adhesion and improved surface characteristics of dental materials. The historical development of CAD/CAM production of PMMA replacements began in the 1990s, when [4] first produced removable dentures using 3D laser lithography [5], improving the duplication of replacements with a CNC system, thus laying the foundations for milling from PMMA blocks. Modern systems such as AvaDent then enabled the milling of denture bases directly from pre-polymerized PMMA blocks, resulting in improved strength, accuracy and a reduced number of patient visits compared to conventional PMMA. The first pilot clinical studies on these blocks [6] confirmed their clinical applicability and advantages in standardization [7]. Between 2010 and 2015, studies such as [8] focused on the combination of cutter type and material, revealing the influence of tool geometry and polymer hardness on cutter wear and surface quality. Since 2015, PEEK and PEKK machining have also been intensively researched, with [9] investigating cutting parameters—feed per tooth, spindle speed, and cutting depth—and their impact on surface roughness and material integrity. During this period, statistical optimization methods such as Taguchi design and Response Surface Methodology began to be used, which made it possible to determine the optimal machining parameters more efficiently. Several studies reported that clinically acceptable roughness values are commonly associated with an appropriate combination of machining parameters and subsequent surface treatment procedures [10]. Current studies, such as [11], have shown that milled PMMA discs exhibit higher flexural strength (105.1 MPa) and mechanical stability than thermally polymerized PMMA (87.9 MPa) and 3D-printed variants thanks to their dense pre-polymerized structure with minimal porosity. For PEEK and PEKK materials, studies emphasize the importance of precise CAD/CAM machining in maintaining mechanical integrity, dimensional stability, and favorable surface quality [12]. With the development of this field, researchers have begun to delve deeper into the specifics of cutting parameters that affect machining performance. This research has revealed that factors such as tool geometry (a smaller cutter diameter of 0.8 mm achieves R_a_ 0.45 µm compared to 1.6 mm: R_a_ 0.78 µm) [13] and feed per tooth (f_z_ = 0.03 mm/tooth for optimal roughness) [14] significantly affect the surface quality of machined biopolymers. The correlation between machining parameters and final surface roughness has become a focal point that has paved the way for machining strategies where cutting speed (v_c_ = 200 m/min) [14] and low feed minimize R_a_ to 0.32 µm for PMMA [15], while high feeds (>300 mm/min) increase roughness due to tool vibration [16,17]. Research into the machining of biopolymers for dental applications has yielded interesting findings, particularly with regard to surface roughness and the specification of cutting parameters. An important topic is the influence of material properties on machining performance, where the elasticity and viscosity of biopolymers (e.g., PEEK, PMMA) directly affect the resulting surface quality during milling [13,18]. Studies using design of experiments systematically analyze the influence of machining parameters such as spindle speed and feed per tooth on the surface roughness and machining performance of dental CAD/CAM polymer materials [14,19]. These optimizations are important for improving the surface quality and manufacturing precision of dental CAD/CAM biopolymer materials, where surface roughness below 0.2 µm may contribute to reduced biofilm adhesion and improved surface characteristics relevant to prosthetic applications [20]. Recent studies have reported similar findings for biopolymers such as PMMA, PEEK, and PEKK. Ref. [8] reports that the surface roughness after milling alone exceeds the clinically optimal limit, and, therefore, additional polishing may be required to achieve values close to R_a_ ≤ 0.2 µm. Similarly, Ref. [6] showed that the adjustment of cutting parameters during PEEK milling can result in surface roughness values close to those commonly reported for dental prosthetic applications. Interesting results were also published in a study from [21], which examined PMMA with ZnO nanoparticles and compared it with milled PMMA. The milled samples showed the lowest R_a_ (0.23 μm), suggesting that CAD/CAM milling may provide a more homogeneous surface than conventional techniques or additive manufacturing. A study published by [22] analyzed the topographical properties of milled surfaces in detail. The authors evaluated several parameters, including R_a_, R_q_, R_z_ and R_sm_, and found that although milling polymers produces a smoother surface than conventional techniques, significant surface roughness remains. This can negatively affect aesthetics and cause wear on opposing teeth, highlighting the need for subsequent surface treatments. Surface quality after milling is considered an important factor influencing composite adhesion, plaque accumulation, aesthetics, patient comfort, and the long-term durability of prosthetic components [10,11]. PMMA is traditionally used for temporary superstructures and provisional crowns, with milling allowing for high shape accuracy and a homogeneous surface. PEEK and PEKK are high-performance biopolymers, whose modulus of elasticity resembles that of human bone, making them ideal for permanent superstructures. However, these materials require a specific approach to milling due to their higher hardness and sensitivity to thermal stress in order to avoid microcracks and surface degradation [9]. Surface roughness after milling is determined by a combination of the chemical composition and hardness of the material, the geometry of the milling tool, the cutting parameters and the use of cooling. Higher spindle speeds combined with low feed rates typically result in a smoother PMMA surface, while for PEEK, cutting parameters need to be optimized to prevent microcracks and surface degradation [9]. Surface roughness also influences the functional behavior and long-term performance of dental prosthetic materials. A smoother surface may contribute to reduced plaque accumulation and improved surface characteristics relevant to dental prosthetic applications. Selected milling parameters in combination with subsequent treatments such as sandblasting or plasma treatment may contribute to improved surface quality and clinically acceptable results. Especially for high-performance polymers and innovative materials such as G-CAM, suitable machining parameters and subsequent surface treatments are important for improving surface quality and dimensional stability. Study [23] demonstrated that milling orientation affects not only the final surface quality but also the degree of internal stress in the material, which may influence its long-term stability. In the case of polymers with high viscoelasticity, such as PEEK, incorrect parameter selection can lead to microcracking or thermal damage to the surface [24]. Study [3] analyzed the milling of CF-PEEK composites (PEEK with carbon fibers) and used grey relational analysis and ANOVA to determine the optimal combinations of cutting speed, feed per tooth and cutting depth that minimize R_a_ and maximize productivity. Study [25] examined the various parameters of milling biocompatible PEEK material and found that the axial depth of the cut has the greatest influence on R_a_, followed by feed rate and the radial depth of the cut. They also pointed out that a noticeably smoother surface can be achieved at lower depths and appropriately selected feed rates. The study “Fundamentals of the milling process of biocompatible PEEK material” focused on the processing of pure PEEK material using carbide cutting tools. The study analyzed the basic principles of the milling process of this biocompatible polymer with the aim of optimizing surface quality and machining efficiency. Another study, “Optimization of PEEK machining parameters” [26], used an optimization approach based on the Response Surface Methodology (RSM) for pure PEEK material. The results showed that the correct choice of cutting speed, feed rate and cutting depth can significantly reduce surface roughness while maintaining high machining efficiency. Overall, these studies suggest that the precise optimization of milling parameters is important for achieving improved surface quality while minimizing tool wear and manufacturing costs during the machining of pure PEEK material. Despite existing research, there is still a lack of systematic studies focused on graphene-reinforced hybrid biopolymer CAD/CAM materials such as G-CAM using a uniform surface roughness evaluation methodology. Previous studies primarily focused on conventional PMMA, PEEK, or PEKK materials, individual machining conditions, or post-processing methods, while limited attention has been devoted to regression-based prediction and comparison of multiple machining parameter configurations under identical experimental conditions. In addition, there is still insufficient standardization of milling parameters for achieving improved surface quality directly after milling without additional polishing operations. The novelty of this study lies in the systematic evaluation of machining parameters during the milling of graphene-reinforced G-CAM PMMA-based biopolymer discs using a full factorial design of experiments approach combined with regression-based prediction of surface roughness. The study compares manufacturer-recommended, average, and optimized cutting conditions in order to identify machining parameters that lead to improved surface quality and reduced surface roughness after milling. The main objective of this study is to analyze the influence of selected cutting parameters on the resulting surface roughness and to identify suitable machining conditions for improving the surface quality of milled biopolymer CAD/CAM materials.

## 3. Materials and Methods

The aim of the presented experimental research is to optimize the technological process of manufacturing dental prosthetic components from biopolymer CAD/CAM materials. The optimization of cutting parameters is focused on improving surface quality after machining while reducing the need for subsequent finishing operations such as mechanical polishing or manual surface treatment. Reducing these additional operations may improve the time, economic, and technological efficiency of production. The primary focus of the research is on determining suitable milling parameters, specifically cutting speed, feed per tooth, and cutting depth, which significantly influence the resulting surface topography. Surface roughness is considered one of the key indicators of machined surface quality, as it may influence plaque accumulation, surface characteristics, aesthetic properties, and the long-term performance of dental prosthetic components. Based on previous studies, surface roughness values of around R_a_ ≤ 0.2 μm are commonly associated with the reduced bacterial adhesion and improved surface characteristics of dental restorative materials.

### 3.1. Material Characteristics

The experiment was performed on G-CAM™ biopolymer discs, which are a modern CAD/CAM material designed for the manufacture of temporary and semi-permanent dental prosthetic components. This material is based on a polymethyl methacrylate (PMMA) matrix with the addition of hybrid components, whose role is to improve the mechanical properties, dimensional stability and wear resistance. Thanks to its homogeneous microstructure and good machinability, this type of biopolymer is suitable for the systematic study of the influence of cutting conditions on surface quality after CNC milling. The samples were milled on a multi-axis CNC milling unit designed for dental CAD/CAM applications, using standard cutters recommended by the material manufacturer. During the experiment, the selected cutting parameters were systematically changed within a predefined experimental plan in order to identify their individual and mutual influence on the resulting surface roughness. The experimental design made it possible to reduce the number of tests while maintaining sufficient statistical significance of the results and ensuring the reproducibility of the measurements. After machining, the surface roughness of the samples was evaluated using a contact profilometer. Measurements were taken at several locations on each sample to eliminate the influence of local surface inhomogeneities and ensure the representativeness of the measured values. The obtained data were statistically processed to determine the optimal combinations of cutting parameters leading to reduced R_a_ without the need for additional polishing operations. The results of this experiment represent a significant contribution to the optimization of technological processes in the field of digital dentistry and can serve as a practical basis for manufacturers of dental prosthetic components when setting production strategies for biopolymer CAD/CAM materials.

**Figure 1:**
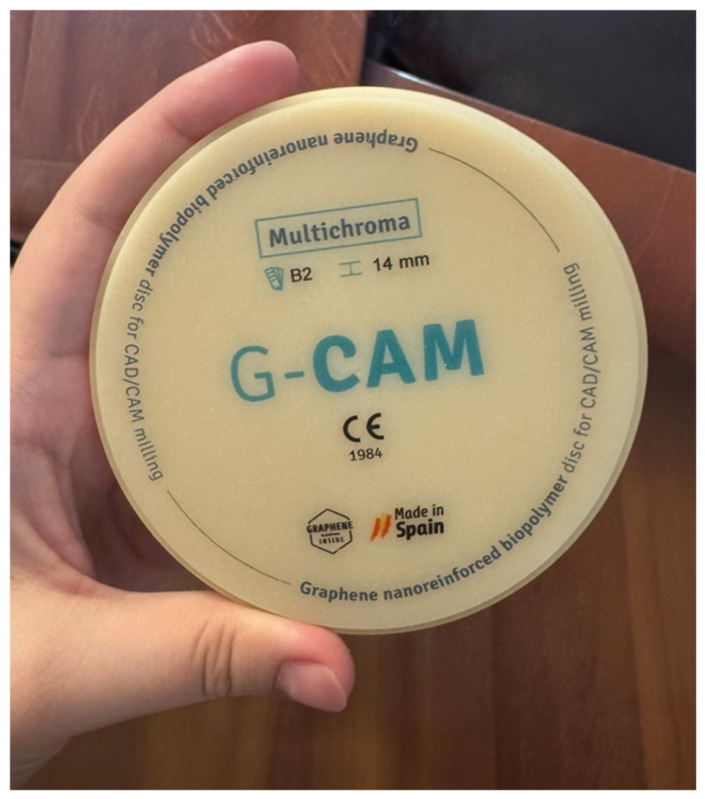
G-CAM biopolymer disc used as the experimental material for the milling experiments.

Figure 1 presents the G-CAM biopolymer disc used as the experimental material for the milling experiments. Table 1 summarizes the key material properties of the G-CAM biopolymer disc These biopolymer discs are characterized by a homogeneous internal structure, increased flexural strength, and a high degree of biocompatibility, which makes them suitable for use in digital dentistry and CAD/CAM prosthetic applications. They are primarily intended for the manufacture of fixed prosthetic replacements, such as individual crowns, bridges, inlays, and onlays, as well as prosthetic restorations manufactured using CAD/CAM milling technologies. Thanks to a controlled manufacturing process and uniform distribution of reinforcing and hybrid components in the PMMA matrix, they exhibit stable mechanical properties throughout the entire volume of the material, which is extremely important in terms of machining accuracy and predictability of results. Compared to conventional PMMA blocks, these hybrid biopolymer discs represent a more technologically advanced solution, primarily due to their higher resistance to the formation and propagation of fractures during functional loading and during machining itself. Increased flexural strength contributes to the better clinical reliability of replacements, especially in more extensive bridge constructions or prosthetic restorations exposed to cyclic chewing loads.

### 3.2. Machining Equipment and Experimental Setup

**Figure 2:**
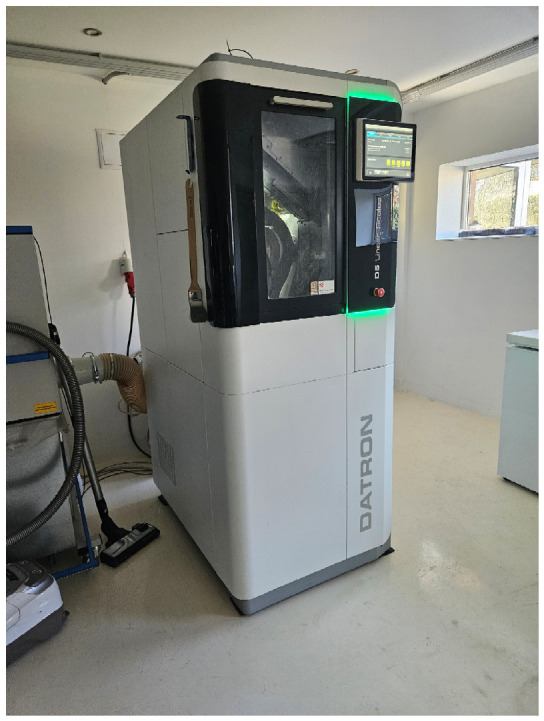
Datron D5 linear scales.

The machining process was carried out using a Datron D5 Linear Scales CNC milling machine (DATRON AG, Mühltal, Germany) designed for high-precision dental CAD/CAM applications [28]. Figure 2 shows the Datron D5 Linear Scales CNC milling machine.The machine enables multi-axis machining with high positioning accuracy and process stability, which is essential for manufacturing polymer-based dental prosthetic components with controlled surface quality and dimensional precision. During the experimental machining process, variations in the main technological parameters were applied in order to evaluate their influence on the resulting surface roughness of the machined material.

In the experimental part, three key parameters for a ∅ 1 mm ball milling DATRON dental milling tools (DATRON AG, Mühltal, Germany) cutter were examined:

- cutting speed (v_c_);
- feed per tooth (f_z_);
- cutting depth (a_p_).

**Figure 3:**
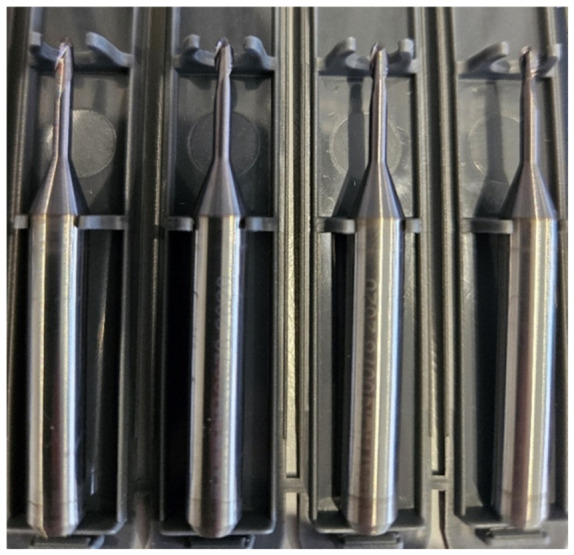
Smoothing ball cutters ∅ 1 mm.

As part of the experimental part of the study, three different combinations of cutting parameters were systematically selected, taking into account practical operating conditions, technological machining possibilities and research objectives focused on analyzing the impact of cutting conditions on the resulting surface quality. Figure 3 shows the smoothing ball cutter (∅ 1 mm) used in the experiments. The selection of machining parameter ranges was based on manufacturer recommendations, preliminary machining tests, and published studies focused on the milling of PMMA- and PEEK-based dental CAD/CAM materials. The experimental methodology followed commonly used procedures for surface roughness evaluation in dental CAD/CAM machining studies reported in the literature. The selection of these combinations was not random—it reflected a strategic approach aimed at covering a wide range of operating situations, with an emphasis on improving surface roughness when machining polymer discs intended for dental prosthetic components. The selected cutting parameters were systematically varied at several levels according to a predefined experimental plan, which allowed for a quantitative assessment of the impact of individual technological factors on the final quality of the machined surface, represented by R_a_. This approach provided the basis for analyzing the main effects and interactions between factors within a multifactorial experiment. All experimental cycles were carried out under consistent conditions, using the same type of cutting tool (same material, geometry, coating) to eliminate the influence of different tool characteristics on the results. This standardization of tools was key to ensuring the reproducibility and objectivity of the data obtained. A stream of compressed air was used to provide cooling during machining, which served to cool and remove chips from the cutting site. This method was chosen with regard to the machined material, which is sensitive to thermal damage and also requires dry or minimal lubrication conditions to prevent surface contamination. The use of compressed air helped to maintain a stable temperature for both the tool and the workpiece while minimizing the risk of microcracks or thermal defects [29].

**Figure 4:**
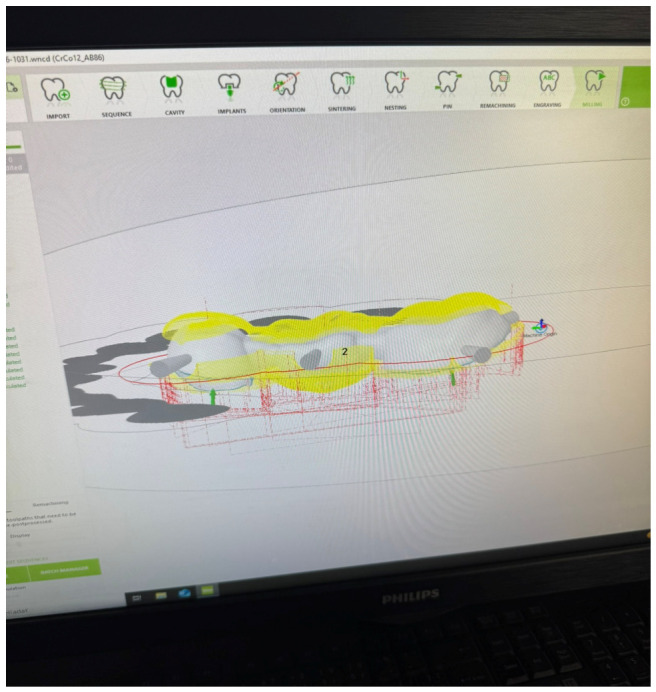
Path generation in WorkNC Dental software.

The results obtained were then processed using statistical methods to determine the significance of the influence of individual parameters (cutting speed, feed per tooth, cutting depth) on the final surface quality. The result of the experiment is the definition of a combination of milling parameters that will allow the desired surface roughness value to be achieved without the need for additional polishing. These findings represent a direct contribution to more efficient production of dental prosthetic components, as they shorten production time and reduce the risk of errors during the manual handling of samples. In the experiment, all samples were machined under identical production process conditions to eliminate the influence of secondary factors. A new tool of the same type was used in each series of measurements to ensure consistency of results and to eliminate possible bias caused by cutting tool wear. The only variables in the experiment were the cutting parameters, which were set at two levels to determine their effect on the surface roughness of the milled polymer discs. Production preparation took place in the WorkNC Dental software 2023 (Hexagon Manufacturing Intelligence, Grenoble, France) environment, which allows the design of digital models of dental prosthetic components and the subsequent generation of code for the Datron D5 machining center [30]. Figure 4 shows the toolpath generation in WORKNC Dental software. This system provides precise control over the entire machining process, including the selection of tool trajectories and the optimization of the cutting strategy according to the type of material being processed. This experimental approach made it possible to systematically investigate the effect of cutting parameter variations on surface quality without the need for further finishing operations.

### 3.3. Surface Roughness Measurement

**Figure 5:**
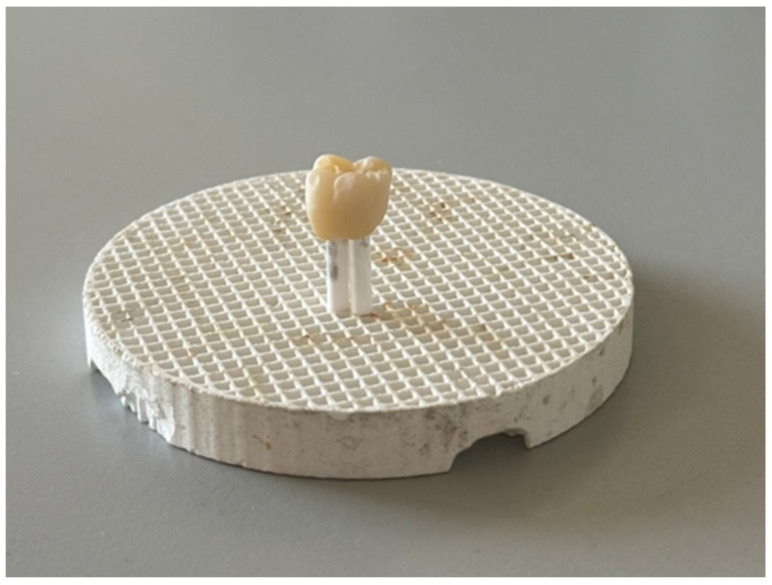
Final sample prepared for measurement.

After milling, the surface roughness of the samples was evaluated using a Mitutoyo Surftest SV-2100 (Mitutoyo Corporation, Kawasaki, Kanagawa, Japan) contact profilometer. Figure 5 shows the final sample prepared for measurement. The arithmetic mean R_a_ was selected as the primary response variable for assessing the quality of the machined surfaces.

Surface roughness measurements were performed in both the X and Y directions at several locations on each machined sample in order to minimize the influence of local surface irregularities and material inhomogeneities. Each reported R_a_ value represents the arithmetic mean of five independent measurements performed on the same sample. This measurement approach increased the accuracy, repeatability, and reliability of the obtained data while reducing the influence of random measurement deviations. The measured roughness values were subsequently used for statistical evaluation and for analyzing the relationship between the machining parameters and the resulting surface quality.

### 3.4. Experimental Design and Statistical Analysis

The experimental design was based on a full factorial design of experiments approach using a 2^3^ factorial configuration. Three machining parameters were selected as independent variables: cutting speed, feed per tooth, and cutting depth. Each parameter was evaluated at two levels, corresponding to the lower and upper limits defined for each experimental series. For each experiment, eight unique parameter combinations were generated from the factorial design. Every combination was repeated three times to ensure the reproducibility and reliability of the measured data, resulting in a total of 24 machining runs per experiment. A total of 72 machined samples were evaluated across the three experimental series. The experiments were performed under identical machining conditions using the same machine tool configuration, identical tool geometry, and the same cooling method. To eliminate the influence of tool wear, a new cutting tool was used for each experimental series. The experimental runs were carried out on a Datron D5 Linear Scales CNC milling machine using a ∅1 mm ball-end milling cutter intended for dental CAD/CAM machining applications. Cooling and chip removal were ensured using compressed air under dry machining conditions in order to avoid thermal degradation and contamination of the polymer surface. Surface roughness evaluation was performed using a Mitutoyo Surftest SV-2100 contact profilometer. The arithmetic mean R_a_ was selected as the primary response variable. For each machined sample, five independent measurements were performed at different locations on the surface in both the X and Y directions. The final R_a_ value used for statistical analysis was calculated as the arithmetic mean of these measurements. To minimize systematic bias, the order of experimental runs was randomized before machining. Statistical processing of the experimental data was performed using Minitab Statistical Software, version 21 (Minitab LLC, State College, PA, USA).The measured data were statistically processed using analysis of variance (ANOVA) and regression analysis to determine the significance of the main effects and interactions between the machining parameters. The significance level for all statistical evaluations was set to α = 0.05. The general regression model used for evaluating the influence of machining parameters on surface roughness can be expressed as follows:

R_a_ = β0 + β1v_c_ + β2f_z_ + β3a_p_ + β12v_c_f_z_ + β13v_c_a_p_ + β23f_z_a_p_ + β123v_c_f_z_a_p_ + ε

where β0 represents the intercept, βi are regression coefficients corresponding to the individual factors and their interactions, and ε represents the random experimental error. In addition to the full regression model, backward regression analysis was applied to eliminate statistically insignificant terms and generate simplified predictive models containing only significant parameters influencing the resulting surface roughness.

## 4. Results and Discussion

This section presents the results obtained from the experimental milling of PMMA-based biopolymer CAD/CAM materials and evaluates the influence of the selected machining parameters on the resulting surface quality. The analysis focused on the effect of cutting speed, feed per tooth, and cutting depth on the machined surface microgeometry represented by the arithmetic mean R_a_. Three experimental series with different machining parameter ranges were analyzed and compared in order to identify conditions leading to improved surface quality. The obtained data were evaluated using statistical methods including analysis of variance (ANOVA), regression analysis, Pareto charts, contour plots, and 3D surface analyses. The three experimental series differed according to the origin and purpose of the selected machining parameters. The first series used cutting conditions recommended by the tool manufacturer, the second series investigated average machining conditions selected within the experimental range, and the third series focused on optimized cutting conditions identified through statistical evaluation of the experimental results.

### 4.1. Manufacturer-Recommended Machining Parameters

In the first experiment, the cutting parameters were selected based on the recommendations of the manufacturer of milling tools used in the production of dental prosthetic components, with minimum and maximum recommended values for cutting speeds, feed per tooth and cutting depth. The aim was to verify whether these standardized conditions would allow surface roughness within a clinically acceptable range to be achieved without the need for additional polishing. Variations in parameters were implemented at two levels—minimum and maximum—while all other process conditions remained unchanged. The experiment prepared in this way provides a baseline for comparison with other series in which different cutting conditions will be examined in order to evaluate their impact on the quality of the resulting surface. The cutting parameters used according to the manufacturer’s recommendations were as follows: cutting speed in the range of 110 to 140 m/min, feed per tooth from 0.04 to 0.08 mm/tooth and cutting depth from 0.2 to 0.3 mm. This approach provides a basic framework for an objective comparison of the influence of different parameter combinations on surface quality and provides a starting point for further experimental investigation. At the same time, however, it was found that these conditions did not lead to entirely suitable surface roughness values, which points to the need to specify cutting parameters.

Based on the factors presented in Table 2, the first experiment was carried out using cutting conditions recommended by the tool and machining system manufacturer. The aim of this experiment was to obtain reference values of the monitored parameters under standard technological machining conditions. The specific parameter settings together with the resulting measured values are presented in Table 3.

**Figure 6:**
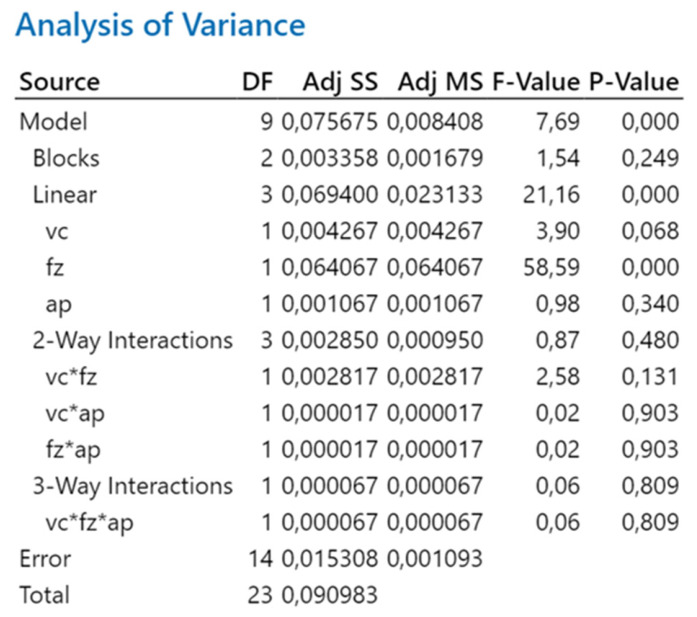
Statistical analysis of variance of the first experiment.

Figure 6 shows the statistical analysis of variance of the first experiment. The analysis showed that feed per tooth (*p* < 0.001) had the most significant influence on R_a_ and represented the dominant machining parameter. Cutting speed was close to the statistical significance threshold (*p* = 0.068), while cutting depth and the evaluated parameter interactions did not show statistically significant effects (*p* > 0.1). The results indicate that proper adjustment of feed per tooth is critical for achieving improved surface quality during the milling of PMMA-based biopolymer CAD/CAM materials.

**Figure 7:**
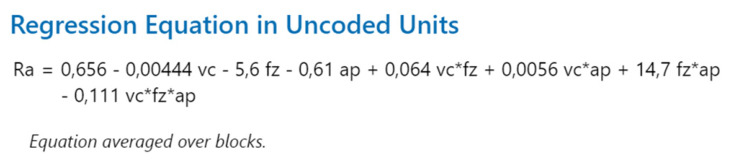
Regression equation of the first experiment.

Figure 7 shows the regression equation of the first experiment. Regression analysis confirmed that feed per tooth had the strongest influence on R_a_, while cutting speed and cutting depth showed lower effects. The interactions between cutting speed and feed per tooth, as well as between feed per tooth and cutting depth, contributed to reduced roughness values. In contrast, three-factor interaction (v_c_*f_z_*a_p_) was associated with increased surface roughness under specific machining conditions.

**Figure 8:**
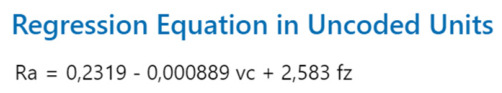
Backward regression equation of the first experiment.

Figure 8 shows the backward regression equation of the first experiment. The simplified regression model for R_a_ was obtained by removing statistically insignificant terms from the original multifactorial regression equation. The final model retained only cutting speed and feed per tooth as significant parameters. Feed per tooth showed the strongest influence on R_a_, with increasing values leading to higher R_a_ values, while increasing cutting speed slightly reduced roughness. The simplified model enables prediction of surface roughness based on the dominant machining parameters while maintaining adequate predictive accuracy.

**Figure 9:**
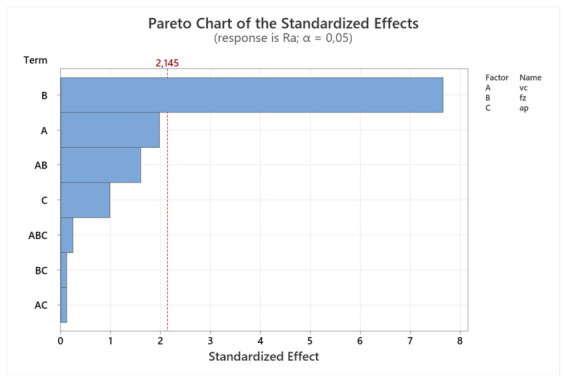
Evaluation of the relative influence of parameters on the result using a Pareto chart in the first experiment.

Figure 9 shows the evaluation of the relative influence of parameters on the result using a Pareto chart in the first experiment. The dashed line indicates the statistical significance threshold (α = 0.05). Pareto analysis identified feed per tooth (factor B) as the dominant parameter affecting surface roughness R_a_. Cutting speed (factor A) and AB interaction showed lower but observable effects, while the remaining factors and interactions remained below the statistical significance threshold.

**Figure 10:**
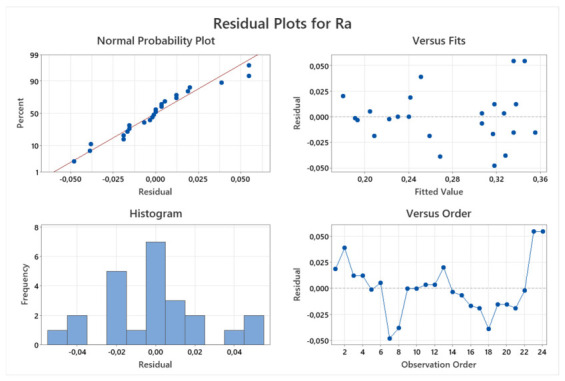
Residual plots of the first experiment.

Residual analysis confirmed the adequacy of the regression model for R_a_, as shown in Figure 10. The residuals showed an approximately normal distribution without significant systematic trends, heteroscedasticity, or autocorrelation, indicating that the model provided an appropriate fit to the experimental data. The normal probability plot further supports the assumption of normality, while the residuals versus fitted values plot confirms constant variance across the range of predicted values.

**Figure 11:**
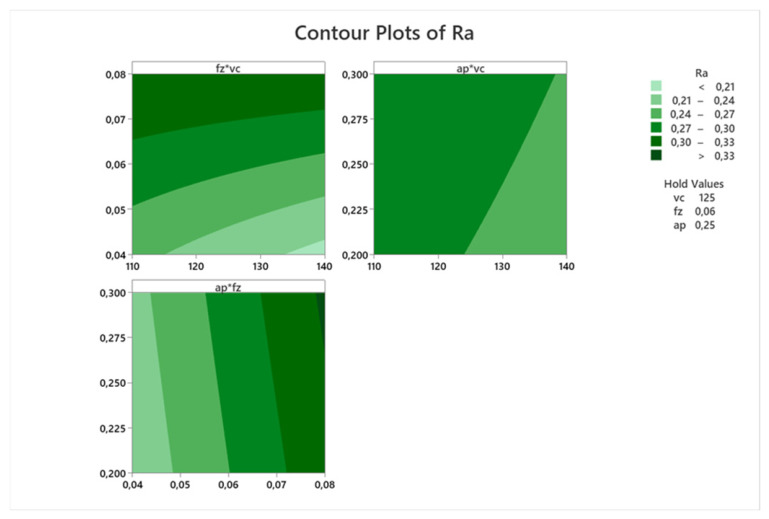
Contour graphs of surface roughness R_a_ from the first experiment.

Contour analysis indicated that lower feed per tooth and lower cutting depth combined with higher cutting speed contributed to reduced surface roughness R_a_ and improved surface quality of the machined PMMA-based biopolymer CAD/CAM materials as shown in Figure 11. The contour lines represent the predicted values of surface roughness obtained from the regression model, while the color scale indicates the magnitude of the Ra parameter.

**Figure 12:**
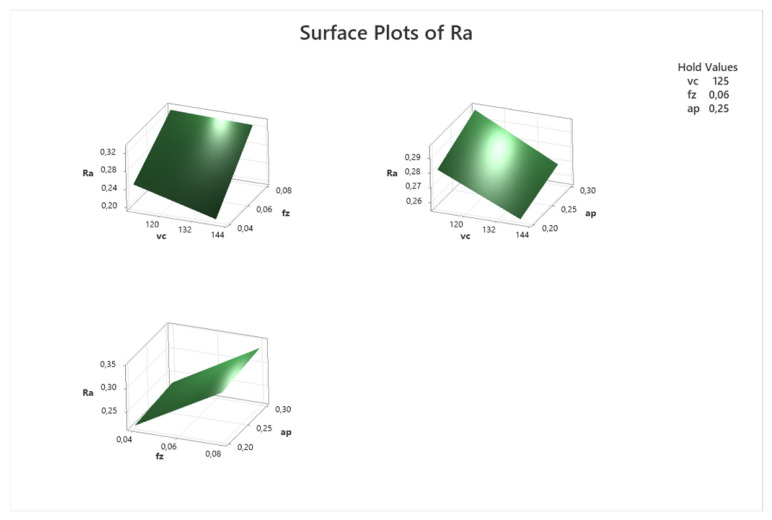
3D surface graphs of surface roughness R_a_ from the first experiment.

The 3D surface analysis, as shown in Figure 12 confirmed that reduced feed per tooth and cutting depth combined with higher cutting speed contributed to lower surface roughness R_a_. The observed trends further demonstrated the significant influence of machining parameters on the resulting surface quality of the milled PMMA-based biopolymer CAD/CAM materials. The colors in the 3D surface graph represent the magnitude of the surface roughness R_a_ parameter, where warmer colors indicate higher Ra values and cooler colors indicate lower Ra values.

### 4.2. Average Machining Parameters

The second experiment was designed analogously to the first, using average cutting parameters for all monitored factors. The aim was to verify how these average settings affect the final surface quality of the material. The experiment allows us to assess whether the application of average cutting conditions provides stable and reproducible results that are clinically acceptable. This approach also determines the extent to which the machining process can be improved without the need for additional surface treatments. The data obtained provide a basis for comparison with experiments performed at minimum and maximum parameter values and help to define the optimal cutting parameters for the subsequent production of dental prosthetic components. The cutting parameters used were set in the following ranges: cutting speeds from 120 to 130 m/min, feed per tooth from 0.05 to 0.06 mm/tooth and cutting depth from 0.2 to 0.3 mm. Analysis of the results showed that the application of average parameter values led to more stable and reproducible surface roughness values, indicating their practical potential in the production of dental prosthetic components. Nevertheless, it was found that these conditions alone do not provide a sufficient level of surface quality, and, therefore, further parameter adjustments are needed to achieve the desired microgeometry, as shown in Table 4.

The second experiment was designed to analyze the behavior of the machining process at average values of the selected cutting parameters. This approach allows the stability of the technological process to be evaluated and the influence of individual parameters on the observed output characteristics to be identified. The overview of parameter settings and the obtained results are presented in the following Table 5.

**Figure 13:**
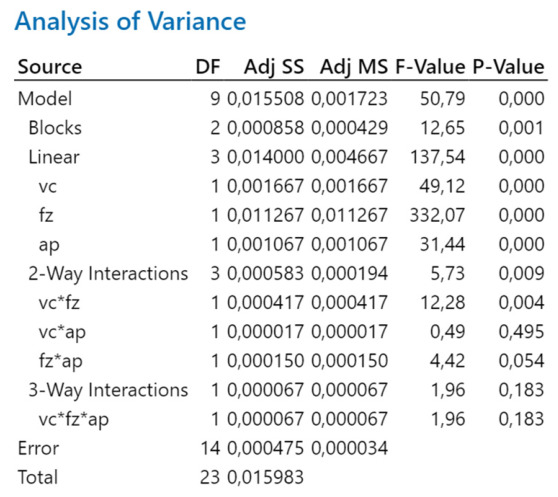
Statistical analysis of variance of the second experiment.

ANOVA analysis, as shown in Figure 13 confirmed that cutting speed, feed per tooth, and cutting depth had statistically significant effects on surface roughness R_a_ (*p* < 0.001). Feed per tooth represented the dominant factor, followed by cutting speed and cutting depth. Among the evaluated interactions, only the interaction between cutting speed and feed per tooth (v_c_*f_z_) showed statistical significance (*p* = 0.004), while the remaining interactions were not statistically significant.

**Figure 14:**
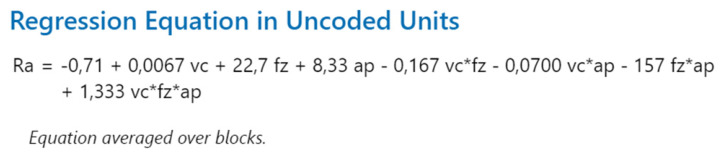
Regression equation of the second experiment.

Regression analysis, as shown in Figure 14, indicated that feed per tooth and cutting depth had the strongest influence on surface roughness R_a_, with increasing values leading to higher roughness. Cutting speed exhibited a lower effect. The interactions between machining parameters generally contributed to reduced roughness values, while three-factor interaction (v_c_*f_z_*a_p_) was associated with increased surface roughness under specific machining conditions.

**Figure 15:**
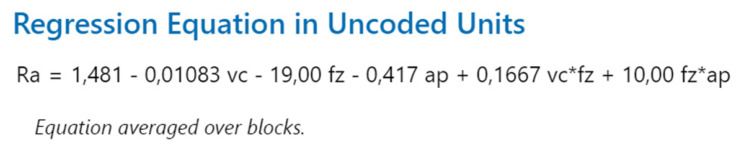
Backward regression equation of the second experiment.

Backward regression analysis, as shown in Figure 15, was used to develop a simplified regression model for surface roughness R_a_ was developed using backward regression analysis, retaining only statistically significant parameters and interactions. Feed per tooth showed the strongest influence on roughness, both individually and through its interaction with cutting depth, while increasing cutting speed slightly reduced R_a_ values. The interaction between cutting speed and feed per tooth indicated that the effect of cutting speed depended on the selected feed conditions. The resulting model provided an improved prediction of surface roughness based on the dominant machining parameters.

**Figure 16:**
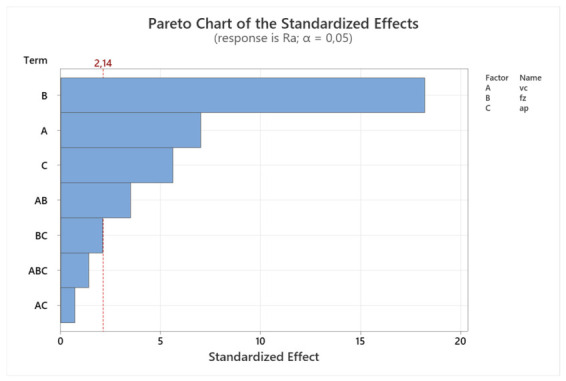
Evaluation of the relative influence of parameters on the result using a Pareto chart in the second experiment.

Pareto analysis, as shown in Figure 16, identified feed per tooth (factor B) as the dominant parameter affecting surface roughness R_a_, with a statistically significant effect above the significance threshold (α = 0.05). Cutting speed and cutting depth showed lower but observable effects, while parameter interactions were not statistically significant. The dashed line in the Pareto chart represents the statistical significance threshold.

**Figure 17:**
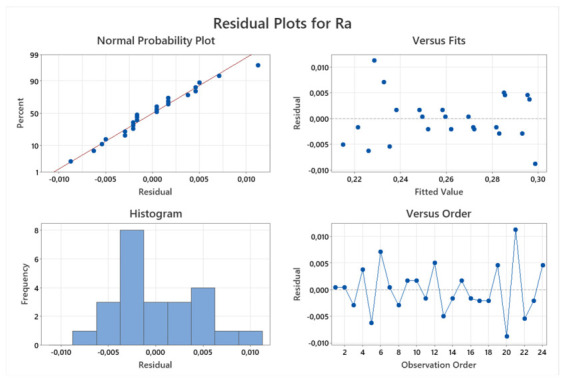
Residual plots of the second experiment.

Residual analysis, as shown in Figure 17, confirmed the adequacy and reliability of the regression model for surface roughness R_a_. The residuals showed an approximately normal distribution without significant systematic trends, heteroscedasticity, or autocorrelation, indicating that the model satisfied the main statistical assumptions and adequately described the experimental data. The normal probability plot further supports the assumption of normality, while the residuals versus fitted values plot confirms constant variance across the range of predicted values.

**Figure 18:**
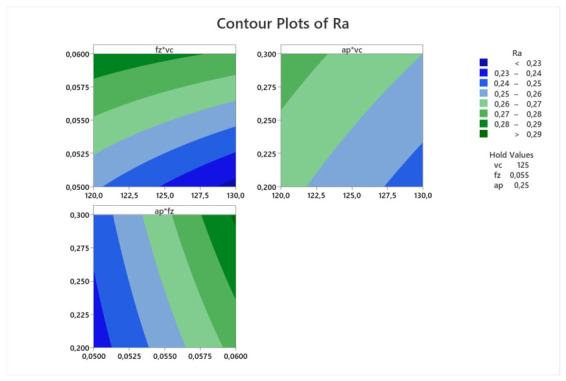
Contour plots of surface roughness R_a_ from the second experiment.

Contour analysis indicated that lower feed per tooth and lower cutting depth combined with higher cutting speed contributed to reduced surface roughness R_a_. Increasing feed per tooth and cutting depth generally resulted in higher roughness values, confirming the significant influence of the machining parameters on surface quality, as shown in Figure 18.

**Figure 19:**
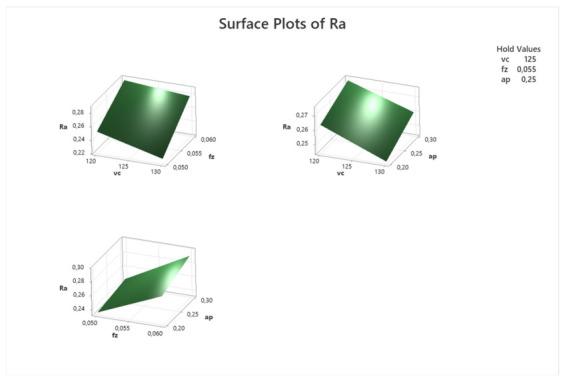
3D surface graphs of surface roughness R_a_ from the second experiment.

The 3D surface analysis confirmed that lower feed per tooth and cutting depth combined with higher cutting speed contributed to reduced surface roughness R_a_. The results consistently demonstrated the dominant influence of feed per tooth on the resulting surface quality, while increasing cutting speed generally reduced roughness values as shown in Figure 19. The color scale in the 3D surface plot represents the magnitude of surface roughness R_a_, with cooler colors indicating lower values and warmer colors indicating higher values.

### 4.3. Optimized Machining Parameters

The third experiment was designed using the most suitable cutting parameter values identified in the previous two experiments. The aim was to verify whether the combination of these settings would achieve minimum surface roughness of the milled biopolymer discs. Unlike the first experiment, where the manufacturer’s recommended parameters were used, and the second, where average values were applied, in this experiment, the values that yielded the best results in the previous measurements were selected. The experiment thus allows us to assess the extent to which the application of these optimal values contributes to achieving the clinically required roughness without the need for additional polishing and to improving the reproducibility of the process results. The cutting parameters used were cutting speeds from 130 to 140 m/min, feed per tooth from 0.04 to 0.05 mm/tooth and cutting depths a_p_ from 0.2 to 0.3 mm. This experiment allowed for a more accurate comparison of the influence of the combination of parameters on surface quality and demonstrated that reducing the feed per tooth in combination with the tested cutting speed leads to the lowest R_a_ values and a homogeneous microstructure in the surface of biopolymer prosthetic restorations, as shown in Table 6.

The third experiment was conducted based on the results obtained from the previous experimental measurements. Based on the analysis of these results, the cutting conditions were modified in order to optimize the monitored machining process parameters. The parameter settings and the obtained experimental results are summarized in the following Table 7.

**Figure 20:**
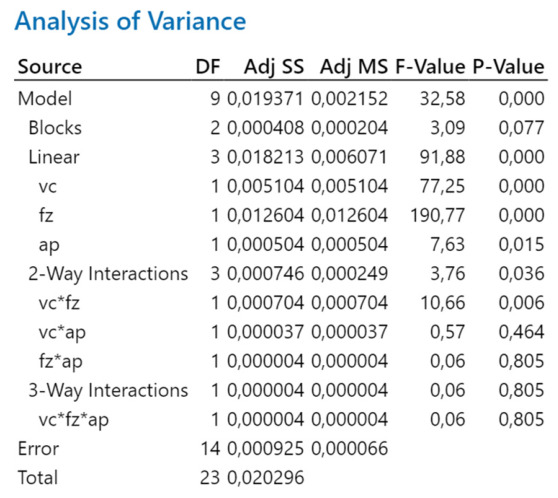
Statistical analysis of variance of the third experiment.

ANOVA analysis, as shown in Figure 20, confirmed that cutting speed, feed per tooth, and cutting depth had statistically significant effects on surface roughness R_a_ (*p* < 0.05). Feed per tooth represented the dominant factor, followed by cutting speed, while cutting depth showed a lower but still significant effect. Among the evaluated interactions, only the interaction between cutting speed and feed per tooth (v_c_*f_z_) was statistically significant (*p* = 0.006), whereas the remaining interactions did not show significant effects on R_a_.

**Figure 21:**
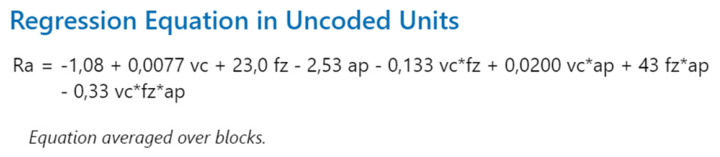
Regression equation of the third experiment.

Regression analysis, as shown in Figure 21, indicated that feed per tooth and cutting depth showed the strongest influence on surface roughness R_a_, while cutting speed exhibited a lower effect. The interaction between cutting speed and feed per tooth contributed to reduced roughness values, indicating improved surface quality under specific machining conditions. In contrast, three-factor interaction (v_c_*f_z_*a_p_) was associated with increased surface roughness.

**Figure 22:**
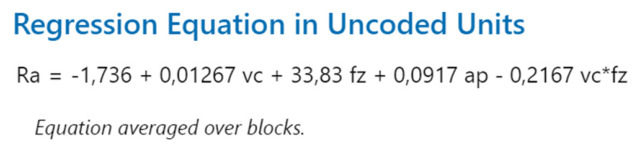
Backward regression equation of the third experiment.

Backward regression analysis, as shown in Figure 22, confirmed that feed per tooth had the strongest influence on surface roughness R_a_, with increasing values leading to higher roughness. Cutting speed and cutting depth showed lower effects, while the interaction between cutting speed and feed per tooth (v_c_*f_z_) partially reduced the increase in roughness caused by feed per tooth. The resulting model described the main linear and interaction effects between machining parameters and supported the prediction of surface roughness during the milling of PMMA-based biopolymer CAD/CAM materials.

**Figure 23:**
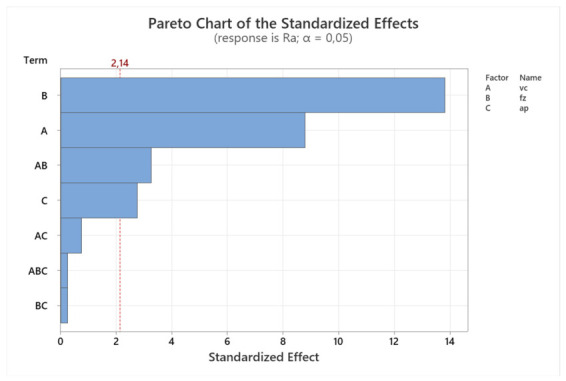
Evaluation of the relative influence of parameters on the result using a Pareto chart in the third experiment.

Pareto analysis, as shown in Figure 23, identified feed per tooth (factor B) as the dominant parameter affecting surface roughness R_a_. Cutting speed (factor A) and the interaction between cutting speed and feed per tooth (A*B) also showed statistically significant effects, while cutting depth exhibited a lower influence on roughness. The dashed line in the Pareto chart represents the statistical significance threshold.

**Figure 24:**
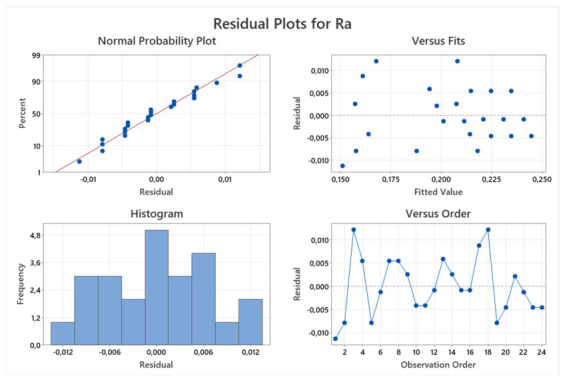
Graphs of residuals from the third experiment.

Residual analysis, as shown in Figure 24, confirmed the adequacy of the regression model for surface roughness R_a_. The residuals showed an approximately normal distribution without significant systematic trends, heteroscedasticity, or autocorrelation, indicating that the model satisfied the main statistical assumptions and accurately described the experimental data. The normal probability plot further supports the assumption of normality, while the residuals versus fitted values plot confirms constant variance across the range of predicted values.

**Figure 25:**
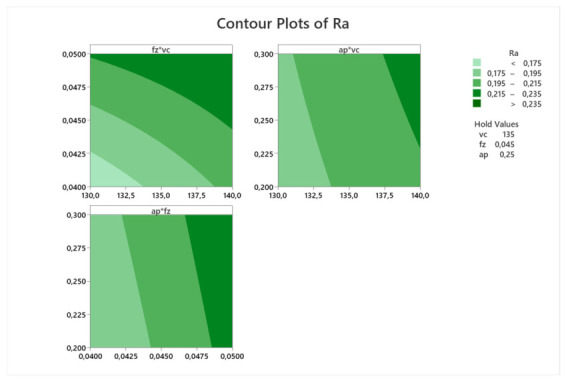
Contour graphs of surface roughness R_a_ from the third experiment.

Contour analysis indicated that lower feed per tooth, lower cutting depth, and lower cutting speed contributed to reduced surface roughness R_a_ and improved surface quality in the machined PMMA-based biopolymer CAD/CAM materials, as shown in Figure 25.

**Figure 26:**
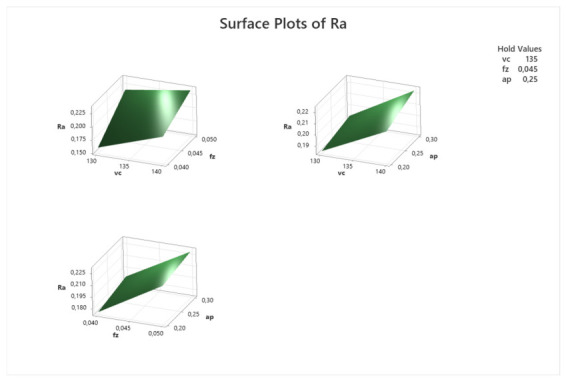
3D surface graphs of surface roughness R_a_ from the third experiment.

The 3D surface analysis confirmed that feed per tooth and cutting depth had a significant influence on surface roughness R_a_. Increasing these parameters generally resulted in higher roughness values, while lower cutting speed contributed to improved surface quality under the evaluated machining conditions, as shown in Figure 26. The color scale in the 3D surface plot represents the magnitude of surface roughness R_a_, with cooler colors indicating lower values and warmer colors indicating higher values.

### 4.4. Discussion of Machining Performance

The obtained results demonstrated that feed per tooth represented the most influential machining parameter affecting the resulting surface roughness of graphene-reinforced PMMA-based G-CAM discs. Lower feed per tooth values were consistently associated with reduced R_a_ values and improved surface quality across all experimental series. The statistical analyses confirmed that optimization of machining parameters significantly influenced machining performance and surface metrology characteristics after milling. The optimized parameter configurations resulted in more stable surface roughness values and reduced variability compared to manufacturer-recommended machining conditions. The observed trends were generally consistent with previous studies focused on the milling of PMMA- and PEEK-based CAD/CAM materials, where feed per tooth and cutting speed were identified as dominant factors influencing surface roughness and machining quality.

## 5. Conclusions

The surface quality of graphene-reinforced PMMA-based G-CAM discs used for dental prosthetic applications was significantly influenced by the selected machining parameters, while R_a_ served as the primary indicator for evaluating machining performance. Three experimental series with different cutting parameter configurations were analyzed and compared in order to evaluate their influence on the resulting surface quality. The first experimental series applied machining parameters recommended by the tool manufacturer and evaluated their lower and upper parameter limits. Although several parameter combinations resulted in relatively low R_a_ values, the obtained results showed higher variability and lower process stability. The second experimental series focused on average machining parameters, which resulted in more homogeneous surface structures and lower average roughness values than the first series. The third experimental series was based on optimized parameter combinations identified from the previous experiments, particularly involving reduced feed per tooth values. This configuration produced the lowest measured R_a_ values and the most stable surface quality among all the evaluated conditions. The results confirmed that feed per tooth represented the most influential machining parameter affecting the resulting surface roughness. Overall, the study demonstrated that appropriate selection and optimization of cutting speed, feed per tooth, and cutting depth significantly influence the resulting surface quality after milling. The obtained results may contribute to process optimization, improved machining efficiency, and the reduction of additional finishing operations in dental CAD/CAM manufacturing applications.

## Figures and Tables

**Table 1:** Technical parameters of the G-CAM discs [27].

Parameter	Value/Description
Base material	PMMA (polymethyl methacrylate) with graphene nano-reinforcement
Color	Translucent, multiple shades according to the VITA shade guide
Flexural strength	120–150 MPa
Modulus of elasticity (E modulus)	3200–3500 MPa
Hardness (Shore D)	approx. 85–90
Water absorption	<0.3%
Wear resistance	Increased thanks to graphene reinforcement
Biocompatibility	Certified, material suitable for permanent and temporary replacements
Indications in dentistry	Crowns, bridges, temporary prosthetic restorations, long-term temporary restorations
Processing technology	CAD/CAM milling (dry)
Thermal stability	Up to 120 °C (short-term higher)
Advantages over conventional PMMA	Higher strength, lower risk of microcracks, lower porosity, better aesthetics

**Table 2:** Determination of factors in the design of the first experiment.

Name	Factor	Unit	−1	+1
X1	Cutting speed v_c_	m/min	110	140
X2	Feed per tooth f_z_	mm/tooth	0.04	0.08
X3	Cutting depth a_p_	mm	0.2	0.3

**Table 3:** Parameter settings and resulting values of the first experiment.

	v_c_	f_z_	a_p_	R_a_
1	110	0.04	0.2	0.26
2	110	0.04	0.3	0.29
3	110	0.08	0.2	0.33
4	110	0.08	0.3	0.35
5	140	0.04	0.2	0.19
6	140	0.04	0.3	0.21
7	140	0.08	0.2	0.27
8	140	0.08	0.3	0.29
9	110	0.04	0.2	0.23
10	110	0.04	0.3	0.24
11	110	0.08	0.2	0.31
12	110	0.08	0.3	0.33
13	140	0.04	0.2	0.20
14	140	0.04	0.3	0.19
15	140	0.08	0.2	0.30
16	140	0.08	0.3	0.30
17	110	0.04	0.2	0.24
18	110	0.04	0.3	0.23
19	110	0.08	0.2	0.32
20	110	0.08	0.3	0.34
21	140	0.04	0.2	0.19
22	140	0.04	0.3	0.22
23	140	0.08	0.2	0.39
24	140	0.08	0.3	0.40

**Table 4:** Determination of factors in the design of the second experiment.

Name	Factor	Unit	−1	+1
X1	Cutting speed v_c_	m/min	120	130
X2	Feed per tooth f_z_	mm/tooth	0.05	0.06
X3	Cutting depth a_p_	mm	0.2	0.3

**Table 5:** Parameter settings and resulting values of the second experiment.

	v_c_	f_z_	a_p_	R_a_
1	120	0.05	0.2	0.25
2	120	0.05	0.3	0.26
3	120	0.06	0.2	0.28
4	120	0.06	0.3	0.30
5	130	0.05	0.2	0.22
6	130	0.05	0.3	0.24
7	130	0.06	0.2	0.27
8	130	0.06	0.3	0.29
9	120	0.05	0.2	0.24
10	120	0.05	0.3	0.25
11	120	0.06	0.2	0.27
12	120	0.06	0.3	0.29
13	130	0.05	0.2	0.21
14	130	0.05	0.3	0.22
15	130	0.06	0.2	0.26
16	130	0.06	0.3	0.28
17	120	0.05	0.2	0.25
18	120	0.05	0.3	0.26
19	120	0.06	0.2	0.29
20	120	0.06	0.3	0.29
21	130	0.05	0.2	0.24
22	130	0.05	0.3	0.23
23	130	0.06	0.2	0.27
24	130	0.06	0.3	0.30

**Table 6:** Determination of factors in the design of the third experiment.

Name	Factor	Unit	−1	+1
X1	Cutting speed v_c_	m/min	130	140
X2	Feed per tooth f_z_	mm/tooth	0.04	0.05
X3	Cutting depth a_p_	mm	0.2	0.3

**Table 7:** Parameter settings and resulting values of the third experiment.

	v_c_	f_z_	a_p_	R_a_
1	130	0.04	0.2	0.14
2	130	0.04	0.3	0.15
3	130	0.05	0.2	0.22
4	130	0.05	0.3	0.22
5	140	0.04	0.2	0.18
6	140	0.04	0.3	0.20
7	140	0.05	0.2	0.23
8	140	0.05	0.3	0.24
9	130	0.04	0.2	0.16
10	130	0.04	0.3	0.16
11	130	0.05	0.2	0.21
12	130	0.05	0.3	0.22
13	140	0.04	0.2	0.20
14	140	0.04	0.3	0.21
15	140	0.05	0.2	0.23
16	140	0.05	0.3	0.24
17	130	0.04	0.2	0.17
18	130	0.04	0.3	0.18
19	130	0.05	0.2	0.21
20	130	0.05	0.3	0.22
21	140	0.04	0.2	0.20
22	140	0.04	0.3	0.21
23	140	0.05	0.2	0.23
24	140	0.05	0.3	0.24

## Data Availability

The original contributions presented in the study are included in the article material, further inquiries can be directed to the corresponding author.

## Abbreviations

The following abbreviations are used in this manuscript:

3D	3-dimensional
ADJ MS	Adjusted mean square
ADJ SS	Adjusted sum of squares
ANOVA	Analysis of variance
a_p_	Cutting depth
CAD	Computer-aided design
CAM	Computer-aided manufacturing
CF-PEEK	Polyether ether ketone with carbon fibers
CNC	Computer numerical control
DF	Degrees of freedom
DoE	Design of experiments
f_z_	Feed per tooth
PEEK	Polyether ether ketone
PEKK	Polyetherketoneketone
PMMA	Polymethyl methacrylate
R_a_	Arithmetic mean surface roughness
R_q_	Mean square deviation of the profile
R_sm_	Average width of profile elements
R_z_	Arithmetic mean of the largest profile heights
v_c_	Cutting speed
ZnO	Zinc oxide
